# No-nonsense: insights into the functional interplay of nonsense-mediated mRNA decay factors

**DOI:** 10.1042/BCJ20210556

**Published:** 2022-05-12

**Authors:** Justine Mailliot, Mirella Vivoli-Vega, Christiane Schaffitzel

**Affiliations:** 1 School of Biochemistry, University of Bristol, University Walk, Bristol BS8 1TD, U.K.; 2 Bristol Synthetic Biology Centre BrisSynBio, 24 Tyndall Ave, Bristol BS8 1TQ, U.K.

**Keywords:** exon-junction complexes, nonsense-mediated mRNA decay, regulation of gene expression, translational control, up-frameshift proteins

## Abstract

Nonsense-mediated messenger RNA decay (NMD) represents one of the main surveillance pathways used by eukaryotic cells to control the quality and abundance of mRNAs and to degrade viral RNA. NMD recognises mRNAs with a premature termination codon (PTC) and targets them to decay. Markers for a mRNA with a PTC, and thus NMD, are a long a 3′-untranslated region and the presence of an exon-junction complex (EJC) downstream of the stop codon. Here, we review our structural understanding of mammalian NMD factors and their functional interplay leading to a branched network of different interconnected but specialised mRNA decay pathways. We discuss recent insights into the potential impact of EJC composition on NMD pathway choice. We highlight the coexistence and function of different isoforms of up-frameshift protein 1 (UPF1) with an emphasis of their role at the endoplasmic reticulum and during stress, and the role of the paralogs UPF3B and UPF3A, underscoring that gene regulation by mammalian NMD is tightly controlled and context-dependent being conditional on developmental stage, tissue and cell types.

## Introduction

Nonsense-mediated messenger RNA decay (NMD) is a conserved pathway in eukaryotes that quality controls mRNAs and also acts to regulate transcript abundance. mRNAs containing a premature termination codon (PTC) are recognised by NMD, which targets them for degradation. Beyond mRNA surveillance, NMD plays an important role in controlling gene expression in mammalian cells, which affects cell cycle regulation, cell viability, response to DNA damage and defence against viral infection [1–6]. Dysregulation of the NMD pathway has been implicated in severe pathologies, including neuro-developmental disorders, cellular stress, and cancer [6–11]. Understanding the molecular mechanisms of NMD is therefore of paramount importance for the development of new treatment strategies aimed at modulating the function and activity of the proteins involved in NMD.

The best understood and characterised NMD substrates are (i) mRNAs containing a long 3′-untranslated region (3′-UTR) which results in a large distance between the terminating ribosome and translation termination-stimulating factors, such as the poly(A)-binding protein (PABP) bound to the 3′-poly(A)-tail [12–17]. (ii) The presence of one or more exon-junction complexes (EJCs) bound downstream of the PTC is a strong stimulator of NMD [18–21]. The EJC is a multi-subunit protein complex which is assembled 20–24 nucleotides upstream of exon–exon junctions during splicing [22]. mRNAs containing PTCs located 50–55 nucleotides upstream of an exon–exon junction are efficiently degraded by NMD. In contrast, normal stop codons are typically located in the last exon, and in this case translation of the mRNAs leads to disassembly of the associated EJCs such that normal mRNAs are EJC-free [23,24]. Remaining EJCs downstream of the PTC result in aberrant translation termination and allow the recruitment of NMD factors to the terminating ribosome which leads to activation of the NMD machinery [25–27].

### Canonical NMD model

In humans, the canonical NMD machinery consists of the conserved up-frameshift (UPF) proteins (UPF1, UPF2, UPF3B), the suppressors with morphological effects on genitalia proteins (SMG1, SMG5, SMG6, SMG7, SMG8 and SMG9) and the EJC, with its subunits eIF4A3, MAGOH, RBM8A (also known as Y14) and CASC3 (also known as BTZ, MLN51), which will be described in detail below.

In the canonical EJC-dependent model for NMD, slowed translation termination at a PTC allows the formation of a ribosome-associated surveillance complex comprising the SMG1–8–9 kinase complex, UPF1, and the eukaryotic release factors eRF1 and eRF3 (SURF) [28]. Interaction of ribosome-bound UPF1 as part of the SURF complex with EJC-bound UPF2 and UPF3B leads to the formation of a decay-inducing complex (DECID) [28,29]. The DECID complex promotes activation of the SMG1–8–9 kinase complex to phosphorylate UPF1. UPF2 activates UPF1's ATPase and helicase functions which are essential for NMD [30,31]. Once activated and phosphorylated, UPF1 triggers the remodelling of the NMD complexes supporting post-termination ribosome recycling via ATP hydrolysis [32] and recruitment of mRNA decay factors to its phospho-sites, including the endonuclease SMG6 and SMG5–7 [33–35]. The SMG5–7 heterodimer interacts with factors responsible for mRNA deadenylation and mRNA 5′-cap removal (DCP2), and the resulting unprotected mRNA ends are then degraded by 5′–3′ exonuclease XRN1 and the exosome complex [33,36–38].

This review will describe recent insights into the molecular mechanisms of canonical NMD factors, with emphasis on structural data, the role of UPF1 isoforms, the function of UPF3B and UPF3A paralogs and the potential impact of EJC composition on NMD pathway choice in mammalian cells.

## The NMD machinery

### Exon junction complexes

**Figure 1.**
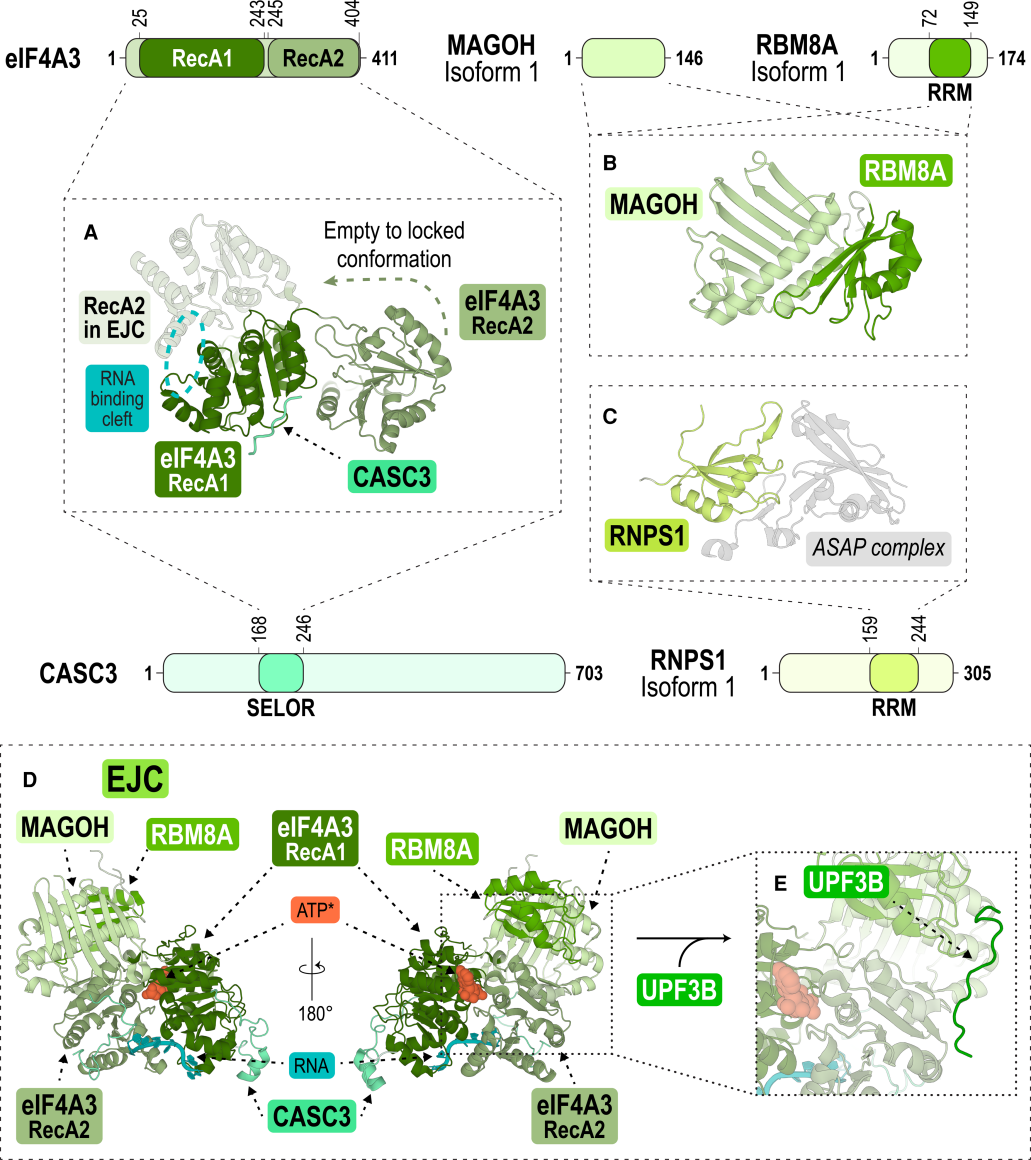
The architecture of the exon-junction complex. Schemes showing the domain architecture of the EJC core proteins eIF4A3, MAGOH and RBM8A, and the EJC-associated proteins CASC3 and RNPS1 which bind in a mutually exclusive manner. In the structures, the protein domains are coloured according to their primary structure scheme. (**A**) Structure of eIF4A3's RecA1 (forest) and RecA2 (pale green) in the presence of CASC3 (aquamarine) only (PDB ID: 2J0U) [43] or in the context of the EJC, in the presence of RNA (cyan circle) and an ATP-analogue (not shown) (PDB ID: 3EX7) [44]. The green arrow indicates the change of eIF4A3's RecA2 domain between open (opaque) [43] and locked (semi-transparent) [44] conformations upon EJC complex formation. (**B**) Structure of the heterodimer of MAGOH (lime) and the RRM domain of RBM8A (green) (PDB ID: 1P27) [166]. (**C**) Structure of the RRM domain of RNPS1 (lemon) bound to the ASAP complex (PDB ID: 4A8X) [167]. (**D**) Front and back view of the EJC bound with an ATP analogue (ATP*, orange), RNA (cyan) and a CASC3 fragment (PDB ID: 3EX7) [44]. (**E**) Close-up view showing UPF3B's EBM bound to the EJC (PDB ID: 2XB2) [49].

EJC core proteins and EJC-associated proteins are intimately connected to mRNA metabolism including its correct splicing, export, mRNA localisation, translation, as well as mRNA turnover including NMD [39,40]. The DEAD-box RNA helicase eIF4A3 is the main RNA-binding component around which the EJC is assembled. eIF4A3 belongs to helicase superfamily 2, which is involved in different RNA-related processes and contains two globular domains (RecA1 and RecA2, Figure 1) with at least 12 characteristic highly conserved sequence motifs, whose functions include adenosine triphosphate (ATP)-binding and hydrolysis (Q, I, II, VI), RNA-binding (Ia, Ib, Ic, IV, IVa, V), and communication between ATP- and RNA-binding sites (III and Va) [41]. Motif II contains the conserved aspartate-glutamate-alanine-aspartate (DEAD) sequence, characteristic of RNA helicases [41]. In the presence of ATP, the two RecA domains adopt a closed conformation that binds the sugar phosphate backbone of six consecutive mRNA nucleotides (Figure 1A,D) [42,43]. MAGOH and RBM8A form a stable heterodimer [44], they lock eIF4A3 onto the mRNA ∼20–24 nucleotides upstream of exon–exon junctions through a tight, hydrophobic, and conserved interaction interface (Figure 1D) [22,42,43,45]. Formation of this EJC core complex inhibits eIF4A3's ATPase and helicase activity [44,46].

MAGOH folds into a single domain formed by six antiparallel β-strands, flanked on one side by two long and one short α-helices (Figure 1B) [42,43]. RBM8A comprises two conserved domains: the N-terminal domain and the RNA-binding-like domain (RBD) essential for MAGOH interaction (Figure 1). The RBD consists of a four-stranded antiparallel β-sheet on one side and two α-helices on the other face (Figure 1B) [42,43]. Atypically, the RBD of RBM8A binds MAGOH and not RNA, with the antiparallel β-sheet of RBM8A packing onto the α-helical surface of MAGOH (Figure 1B) [42].

For a long time, CASC3 (cancer susceptibility candidate gene 3) was considered the fourth EJC core component because it stably interacts with eIF4A3 (Figure 1A,D) and because it is required for EJC assembly *in vitro*. More recently, the role of CASC3 has been revisited because the absence of CASC3 does not affect EJC assembly during its early stages in the nucleus [47]. Based on this, CASC3 was suggested to function as a peripheral EJC protein, required for the degradation of EJC-dependent NMD substrates. Notably, while the EJC composition and EJC-dependent splicing remain virtually unchanged in the absence of CASC3, hundreds of alternatively spliced mRNAs targeted by NMD are up-regulated in CASC3 CRISPR–Cas9 knockout cells [47]. In agreement, when bound to reporter mRNAs by tethering it to the 3′-UTR or via complexing with the EJC, CASC3 stimulates SMG6-dependent endonucleolytic cleavage at the termination codon and thus promotes NMD [47]. Notably, the SMG6 endonuclease contains two conserved EJC-binding motifs (EBMs) and can directly associate with the EJC [48]. Taken together, CASC3 was shown to affect the efficiency by which NMD-sensitive mRNA isoforms are degraded [47].

UPF3B associates with the EJC in the nucleus and is exported with the spliced EJC-bound mRNA, while UPF2 is recruited by UPF3B to the EJC at the nuclear envelope after export [49]. Recent work suggests a role of CASC3 in promoting the association of UPF3B with the EJC and in enhancing UPF3B-dependent NMD [50,51]. This agrees with reports of a link between CASC3-containing EJCs and UPF3B [39,47]. Consistently, in CASC3 knockdown cells, UPF3B's association with the EJC was reduced, and *vice versa* CASC3 overexpression led to enhanced EJC–UPF3B association in HeLa cells [51]. The structure of EJC–UPF3B indicates that the interactions of CASC3 and UPF3B with the EJC core are limited to small regions of the two proteins (Figure 1E) [49,52]. However, it is possible that other, less-structured regions of CASC3 and UPF3B also contribute to their EJC association. Using co-immunoprecipitation (co-IP) and mass spectrometry analyses, Wallmeroth and colleagues reported that CASC3 also interacts with UPF2 in wild-type as well as in UPF3B knockout cells [50]. In conclusion, it appears that CASC3 is a major hub for NMD factor-binding to the EJC.

In addition to CASC3 and UPF3B, the core EJC complex is a dynamic binding platform for other peripheral factors, including the apoptosis- and splicing-associated protein complex (ASAP) composed of SAP18, RNPS1 and Acinus [53–55]. Of these, RNPS1 (RNA-binding protein with serine-rich domain 1) [56,57] has been shown to be able to activate NMD. In the nucleus, RNPS1 works together with the U1 and U2 snRNPs in splicing site recognition. RNPS1's serine-rich region at the N-terminus has been shown to be essential for NMD activation [58], and is followed by an RRM (RNA Recognition Motif) domain and a region containing serine- and arginine-rich repeats at the C-terminus (Figure 1C). Consistently, when tethered to the 3′-UTR of an mRNA [53] or when associated to the EJC during splicing [59,60], RNPS1 promotes mRNA decay.

### Up-frameshift factor 1

**Figure 2.**
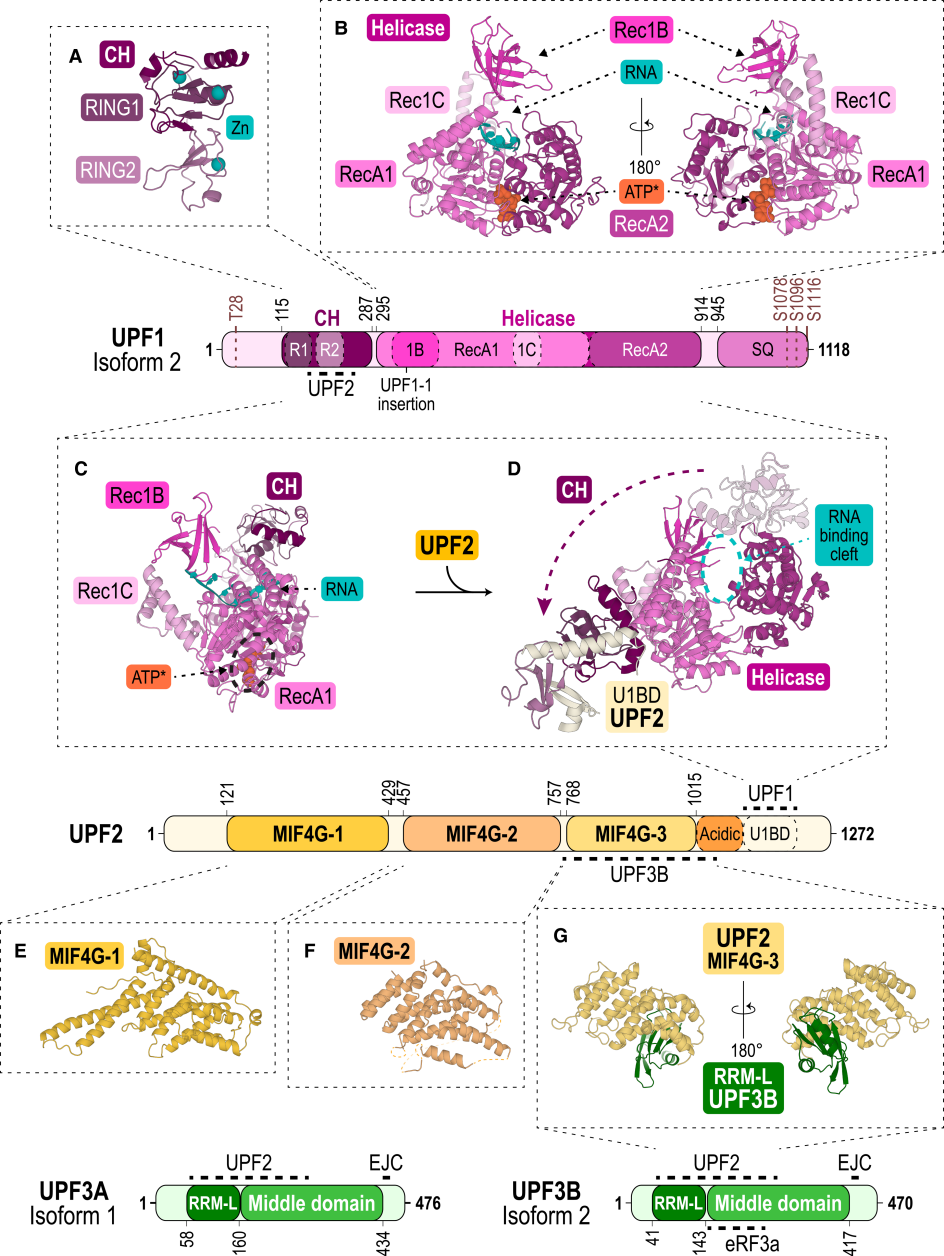
The core NMD factors UPF1, UPF2 and UPF3. Schemes showing the domain architecture of UPF1, UPF2, UPF3B and its paralog UPF3A. In the structures, the protein domains are coloured according to their primary structure scheme. (**A**) Structure of UPF1's CH domain (deep purple), harbouring two RING modules (dark and light violet) (PDB ID: 2IYK) [65] coordinating three Zn^2+^ ions (cyan). (**B**) Front and back view of UPF1's helicase domain with two tandem RecA1 (pink) and RecA2 (dark pink) domains and the subdomains Rec1B (magenta) and Rec1C (light pink) bound with an ATP-analogue (ATP*, orange) and RNA (cyan) (PDB ID: 2XZO) [31]. (**C**) Structure of the yeast CH-helicase domains bound with an ATP-analogue (ATP*, orange) and RNA (cyan) (PDB ID: 2XZL) [31]. (**D**) Structure of the human CH-helicase domains of UPF1 bound with the U1BD of UPF2 (light yellow) (PDB ID: 2WJV) [67]. The arrow indicates the conformational change of the CH domain upon UPF2-binding. (**E**) Structure of UPF2 MIF4G-1 domain (PDB ID: 4CEM, yellow) [87]. (**F**) Structure of MIF4G-2 domain (PDB ID: 4CEK, pale orange) [87]. (**G**) Front and back view of the complex formed by the MIF4G-3 domain of UPF2 (pale yellow) and the RRM-L of UPF3B (green) (PDB ID: 1UW4) [86].

Up-frameshift factor 1 is considered as the key NMD factor. It can bind non-specifically to mRNA and is displaced by the ribosome during translation from the coding sequence, but not from the 3′-UTR [61–64]. UPF1 is a ∼125 kDa protein which is highly conserved in eukaryotes. UPF1 harbours three major domains: a cysteine-histidine-rich domain (CH) and a helicase domain whose structures are well-characterised (Figure 2A,B) [31,65–68], while the serine-glutamine-rich domain (SQ domain) is found within the unstructured C-terminus. UPF1 is hyperphosphorylated by the SMG1–8–9 kinase complex in its disordered N-terminal region (T28) and in its SQ domain (S1078, S1096, S1116 among others) [69]. Recruitment of SMG6 by phospho-UPF1 occurs thanks to interactions with the helicase and C-terminus of UPF1, while SMG5–7 bind to the C-terminal phosphorylated SQ domain [70–72].

Structural characterisation of UPF1's CH domain showed that it forms a unique concatenation of two RING-box like modules (RING1 and RING2) (Figure 2A) [31,65]. The CH domain of UPF1 coordinates three structural zinc ions bound by two zinc-finger motifs located in RING1, and one zinc-finger motif located in RING2 (Figure 2A). Biochemical studies indicate that Upf1 has E3 ubiquitin ligase activity in yeast where it can self-ubiquitinate in the presence of Upf3 and the E2 ubiquitin conjugating enzyme Ubc3 [73]. Furthermore, human UPF1 has been shown to be responsible for the down-regulation of the transcription factor MYOD, a key regulator of myogenesis, not by affecting its mRNA stability, but by promoting its ubiquitination and subsequent degradation by the proteasome [74]. However, it remains to be shown if UPF1 is the E3 ubiquitin ligase directly responsible for MYOD ubiquitination [74]. To date, no other potential targets of the UPF1 E3 ubiquitin ligase have been identified. It is tempting to speculate that UPF1's E3 ubiquitin ligase activity could play a role in the degradation of C-terminally truncated and potentially misfolded polypeptides encoded by nonsense mRNAs. Notably, in other translational quality control pathways, stalled ribosomes trigger poly-ubiquitination of the nascent polypeptide by the E3 ubiquitin ligase LTN1/ Listerin for subsequent degradation by the proteasome [75,76]. However, the fate of the truncated polypeptides produced from PTC-containing mRNAs is currently unknown. Recently, a reporter system with normal and PTC-containing mRNAs encoding the same polypeptide showed specific destabilisation of the protein encoded by the nonsense mRNA which could be overcome by proteasome inhibition [77]. In support of this finding, whole genome knockdown and knockout screens identified the RING E3 ubiquitin ligase CNOT4 (CCR4–NOT transcription complex subunit 4), as a potential enzyme for ubiquitination of the nonsense protein, linking NMD to proteasome-mediated degradation of the encoded nascent polypeptide [78].

The helicase domain of UPF1 harbours the seven motifs distinctive of the DNA/RNA helicase superfamily 1 [30]. To date, several structures of the human UPF1 helicase domain have been solved in different states: in its apo-form [31], in its phosphate-, ADP- (adenosine diphosphate) or AMPPNP-(non-hydrolysable ATP analogue) bound forms [66], as well as in the presence of mRNA in a transition state of ATP hydrolysis with ADP:AlF_4_^-^ [31]. The large array in conformations of the helicase domain highlights the dynamics of the RNA-binding and helicase activities of UPF1. The helicase domain comprises two tandem RecA-like modules (RecA1 and RecA2), with additional β-barrel and α-helical subdomains (Rec1B and Rec1C respectively) which stick out of RecA1 (Figure 2B). Rec1B is connected to RecA1 by a stalk formed by two α-helices. RecA1 and RecA2 face each other, forming a deep ATP-binding cleft [66], and the interface between RecA1, RecA2, Rec1B and Rec1C shapes a large RNA-binding channel (Figure 2B). In the presence of ATP (or ATP analogues), RNA-binding is impaired because of a constriction of the RNA-binding channel, indicating an allosteric effect of this nucleotide, whereas in the presence of ADP the channel becomes wider [30]. These findings corroborate previous observations where ATP-binding was hindering the binding of RNA [79] and ATP hydrolysis was required for UPF1's activity in NMD [80].

In a structure of yeast Upf1, a longer RNA-binding channel formed by the association of the CH and helicase domains was observed [31]. This is achieved by the presence of interactions between the CH domain RING1 module and the helicase stalk, and between the CH domain RING2 module and the helicase RecA2 module (Figure 2C). Thus, the CH domain is responsible for UPF1's improved RNA-clamping and lower RNA-unwinding activity [30]. This is further corroborated by the structure of UPF1 in complex with the C-terminus of UPF2, which illustrates how UPF2 activates UPF1's helicase activity (Figure 2D) [31,65,67]. Upon complex formation with the CH domain of UPF1, UPF2's UPF1-binding domain (U1BD) forms an α-helix that displaces the CH domain from the RecA2 module and a β-hairpin that binds the solvent-exposed side of UPF1's CH domain (Figure 2D) [31,65]. This displacement results in RNA-clamping being reduced and RNA-unwinding by UPF1's helicase activity being stimulated [31,67]. Taken together, these structural studies highlight the importance of the CH domain for the regulation of RNA-binding and helicase activity of UPF1.

In the cell, UPF1 isoform 2 (UniProtKB: Q92900-2) is the most abundant isoform and accordingly has been studied extensively. More recently however, interest in the less abundant isoform 1 of UPF1 (UniProtKB: Q92900-1) has increased [68,81]. The two UPF1 isoforms exist in mammals only and result from the usage of an alternative 5′ splice site in exon 7 of the *Upf1* gene [68]. This alternative splicing adds 11 residues to a loop located in the Rec1B module of the helicase, resulting in UPF1 isoform 1 having a longer loop (UPF1_LL_) while UPF1 isoform 2 has a shorter loop (UPF1_SL_) (Figure 2). This loop plays an important role as it contributes to the RNA-binding channel and thus could regulate UPF1's RNA-binding and helicase activity differently in the two isoforms [68,82]. The shorter loop of UPF1_SL_ is well-ordered in the absence of RNA and points towards the helicase core [31,66]. In the presence of RNA, this short loop is disordered and would likely clash with the bound RNA without a conformational change [31]. The longer regulatory loop of UPF1_LL_ is partially disordered in the absence of RNA and points towards the solvent instead of towards the helicase core [68]. In this conformation, the longer regulatory loop would not interfere with RNA-binding. This is corroborated by studies showing that UPF1_LL_ has higher RNA-binding affinity than UPF1_SL_ in the presence of ATP [68]. While the length of the regulatory loop does not influence ATP-binding directly, it has been shown that UPF1's RNA-dependent ATPase activity is higher when this loop is either replaced with a glycine-serine-rich linker or extended (as in UPF1_LL_) [68]. Consistently, UPF1_LL_ has higher RNA unwinding and translocation activities than UPF1_SL_ [68]. Current findings indicate distinct roles for UPF1_LL_ and UPF1_SL_ in NMD [81]: protective RNA-binding proteins, such as polypyrimidine tract-binding protein-1 (PTBP1) or heterogeneous nuclear ribonucleoprotein L (hnRNP L), were shown to bind to mRNAs with long 3′-UTR and to inhibit translocation of UPF1_SL_ helicase along the mRNA, thus promoting UPF1_SL_ dissociation [83–85]. Because of its higher RNA-binding and unwinding activity, UPF1_LL_ helicase is suggested to overcome this NMD-inhibition by protective RNA-binding proteins and thus promote (EJC-independent) NMD of mRNAs with very long 3′-UTRs [81].

### Up-frameshift factor 2

Up-frameshift factor 2 is a conserved 1272 amino acid protein (∼148 kDa) containing three middle portion of eukaryotic initiation factor 4G domains (three MIF4G domains), flanked by N-terminal and C-terminal regions that are predicted to be inherently unstructured (Figure 2) [67]. The C-terminus harbours an aspartate- and glutamate-rich region, referred to as ‘acidic domain’, followed by the U1BD (Figure 2D). UPF2 is considered a scaffolding protein for the UPF-EJC complex, bringing together UPF1 via the U1BD (see above) and the EJC-bound UPF3B through interactions with its U1BD and third MIF4G domain (MIF4G-3), respectively [52,65,67,86]. Complex formation between UPF2 and UPF1 modulates UPF1's RNA-binding and promotes its ATPase and helicase activities (see above). Additionally, the interaction between UPF2 and the SMG1–8–9 complex is suggested to activate the SMG1 kinase to phosphorylate UPF1 [28,87].

Structures of UPF2's MIF4G-1 and MIF4G-2 domains have been solved separately (Figure 2E,F) [87,88], and the structure of its MIF4G-3 domain was solved in complex with UPF3B (Figure 2G) [86,89]. The domains adopt the canonical MIF4G fold comprised of ten core α-helices arranged into an N-terminal four α-helix bundle that interacts with two layers of three α-helices (Figure 2E–G). Moreover, UPF2's MIF4G-1 domain is stabilised by two additional N-terminal capping α-helices and an additional internal α-helix. Two of the core helices are also highly elongated forming a long, protruding coiled coil (Figure 2E) [87]. UPF2's MIF4G-2 domain displays an N-terminal extension forming two short capping α-helices associated with a third α-helix via a flexible linker (Figure 2F) [87]. The MIF4G-3 domain exhibits the canonical fold and has only one additional C-terminal α-helix (Figure 2G) [86]. In a low-resolution co-crystal structure of UPF2's MIF4G-2 and MIF4G-3, these domains are oriented perpendicularly [87]. Furthermore, low-resolution electron microscopy data indicate that UPF2's three MIF4G domains adopt a ring- or horseshoe-like structure [52,90].

UPF2's MIF4G-3 domain interacts with the RNA recognition like-motif domain (RRM-L) of UPF3B (Figure 2G), allowing the association of UPF2 with the EJC [49,86]. Notably, UPF2 has also been reported to bind the SMG1–8–9 kinase complex and eRF3 [28,87,90], with the MIF4G-3 domain of UPF2 being involved in both interactions. Interestingly, MIF4G-3-binding to eRF3 and to UPF3B are mutually exclusive [90]. In contrast, UPF2 can interact with the SMG1–8–9 kinase complex and UPF3B simultaneously to form a large complex *in vitro*, thereby activating SMG1 kinase activity [87]. A high-resolution structure of SMG1–8–9 in complex with UPF1 and UPF2 is required to shed light on the molecular mechanisms of SMG1 kinase activation by UPF2.

### Up-frameshift factors 3A and 3B

UPF3 exists as two distinct paralogs, UPF3A and UPF3B, which are the products of an ancestral gene duplication event and are believed to be functionally distinct. UPF3B is considered as the main paralog in NMD and is better characterised than UPF3A. UPF3B comprises an N-terminal conserved RRM-L domain which lacks critical aromatic residues required for high-affinity RNA-binding by RRMs [86]. Accordingly, UPF3B's RRM-L only weakly interacts with RNA [86,89]. Instead, UPF3B binds the MIF4G-3 domain of UPF2 via the same binding surface which is used by other RRM's to bind RNA (Figure 2G) [86]. The RRM-L is followed by UPF3B’s middle-domain and a C-terminal EJC-binding motif (EBM) (Figure 2). The EBM of UPF3B interacts with the EJC core via the MAGOH–RBM8A heterodimer and via the RecA2 domain of eIF4A3, which remain virtually unaltered upon UPF3B-binding (Figure 1E) [49].

The middle-domain of UPF3B is poorly characterised. This part is predicted to be largely unstructured having a high density of positively charged residues, which may form two long coiled coil-like helices [89]. Biochemical characterisation indicated that the middle-domain of UPF3B is involved in binding UPF1, eRF3, RNA and ribosomes [91]. Moreover, a recent co-crystal structure of the MIF4G-3 domain of UPF2 in complex with the UPF3B's RRM-L followed by the N-terminal part of its middle-domain, revealed an intimate interaction between the two NMD factors, highlighting that the middle-domain of UPF3B contributes to UPF2-binding. In the structure, UPF2's MIF4G-3 domain is wedged between the RRM-L and the middle-domain's N-terminal portion, which adopts a NONA/paraspeckle-like linker followed by an α-helix [89]. This domain architecture is homologous to the family of paraspeckle (Drosophila behaviour/ human splicing) proteins [92], which serve as functional aggregators of nucleic acids in the nucleus.

The presence of the N-terminal portion of the middle-domain results in a significantly tighter interaction (∼200-fold) between UPF3B and UPF2 compared with the RRM-L alone [86,89]. Importantly, the structure of the UPF2-3B complex comprised UPF3B residue Y160 which was reported to be mutated to aspartate (Y160D) in patients with neurodevelopmental disorders [93,94]. UPF3B's middle domain residue Y160 is buried in a hydrophobic cleft formed by UPF2's MIF4G-3 domain. The Y160D mutation results in a ∼40-fold reduction in UPF2-binding affinity, as this residue is likely is displaced from UPF2's hydrophobic cleft due to its charge [89]. The finding that Y160 is phosphorylated in human cells highlights post-translational modification of UPF3B as a means to weaken complex formation with UPF2 and thus down-regulate NMD efficiency [89].

In patients with the UPF3B Y160D mutation, an up-regulation of the UPF3A paralog has been reported [94]. Similarly, knockout/ knockdown of UPF3B leads to increased levels of UPF3A [95,96]. UPF3A shares the domain architecture of UPF3B but its EBM has lower binding affinity to the EJC (Figure 2) [97]. In an UPF3B-deficient context, UPF3A has been shown to be an active NMD factor that can compensate for UPF3B [50,51,97] while in other contexts, UPF3A can function as a NMD inhibitor, protecting mRNAs from NMD [98] (see below).

Recently, Yi and colleagues observed that UPF3A can replace UPF3B in UPF3B knockout cells and then acts as a NMD activator on a similar set of mRNAs [51]. However, the use of a β-globin reporter mRNA with a PTC at codon 39 demonstrated that overexpression of UPF3A could not rescue NMD in the UPF3B knockout cells [51]. Interestingly, when the middle-domain of UPF3A was replaced with UPF3B's middle-domain, NMD was rescued for the β-globin reporter [51]. However, the molecular basis governing this functional difference in the middle-domains of the two paralogs remains to be explored. Consistently, Wallmeroth and colleagues found that UPF3A could compensate for the loss of UPF3B, and keep NMD activity virtually unaltered, while depletion of both UPF3 paralogs resulted in NMD inhibition and up-regulation of PTC-containing mRNAs [50].

UPF3B is proposed to function as a bridge between the EJC and the SURF complex in the EJC-dependent NMD model [30,50,51,97]. In contrast, the EBM of UPF3A has a weak affinity to the EJC and does not appear to be stably associated with the EJC [50,51,97], suggesting that UPF3A can activate NMD independently of the EJC.

Similarly, rescue experiments with UPF3B variants, performed in UPF3-double knockout cells, suggest UPF3B functions of which do not require EJC-SURF bridging [50]: the EBM or the UPF2-binding site in the RRM-L can be depleted or mutated, respectively, without affecting NMD efficiency. NMD was only impaired in the case of double mutants of RRM-L and EBM. Moreover, NMD was also inhibited if the middle domain of UPF3B was deleted in addition to deletion of the EBM or to mutation of the RRM-L [50]. Taken together, these findings highlight a role of UPF3B beyond bridging the EJC and the ribosome-bound SURF complex.

A possible EJC-independent function of UPF3B has been reported previously using a reconstituted mammalian translation system [91]. In this *in vitro* system, addition of UPF3B was shown to interfere with stop codon recognition and peptide release from the ribosome, thus reducing the efficiency of translation termination. In agreement with a role during translation termination at a PTC, UPF3B has been shown to bind eRF3a, UPF1 and ribosomes in pulldown and co-sedimentation experiments [91]. It was shown that the RRM-L and the middle-domain are required, and that the EBM is dispensable for UPF3B's inhibitory effect on translation termination [91].

In the presence of the EJC, Hauer and colleagues suggested that UPF3B participates in the positioning of the EJC and the NMD machinery, based on iCLIP experiments, where UPF3B was found to bind both the EJC core and mRNA [99].

An intriguing picture emerges where UPF3B and UPF3A levels fine-tune NMD efficiency and thus the transcriptome of cells. The relative and absolute levels of the UPF3 paralogs differ during development and in different cell types and tissues [98,100]. The levels of both paralogs appear to be tightly regulated and are of paramount importance during neurodevelopment, as evidenced by UPF3B mutations leading to decreased NMD efficiency and neurodevelopmental disorders in affected families [93,96]. For instance, in the case of the UPF3B Y160D mutation (see above), a slightly reduced NMD efficiency causes an up-regulation of nonsense mRNAs encoding the ATF4 transcription factor and the ARHGAP24 signalling factor. The consequence of this are changes in neuronal differentiation and a reduction in neurite branching, despite the up-regulation of UPF3A to compensate for the loss of UPF3B function [93,94]. Future work is required to shed light on the roles of UPF3B in translation termination and NMD, and to identify the functional differences between the paralogs UPF3A and UPF3B.

### The SMG1–8–9 complex (SMG1C)

SMG1 kinase (∼410 kDa) belongs to the family of phosphatidylinositol-3-kinase-related kinases (PIKKs) [101]. SMG1 phosphorylates UPF1 at serine/threonine-glutamine (SQ) motifs present in the unstructured N- and C-terminal regions that flank the CH-helicase core (Figure 2) [102]. In addition, several UPF1 phosphorylation sites have been identified sharing a leucine-serine-glutamine (LSQ) consensus sequence which is identical with that found in ataxia telangiectasia mutated (ATM) kinase substrates [103].

**Figure 3.**
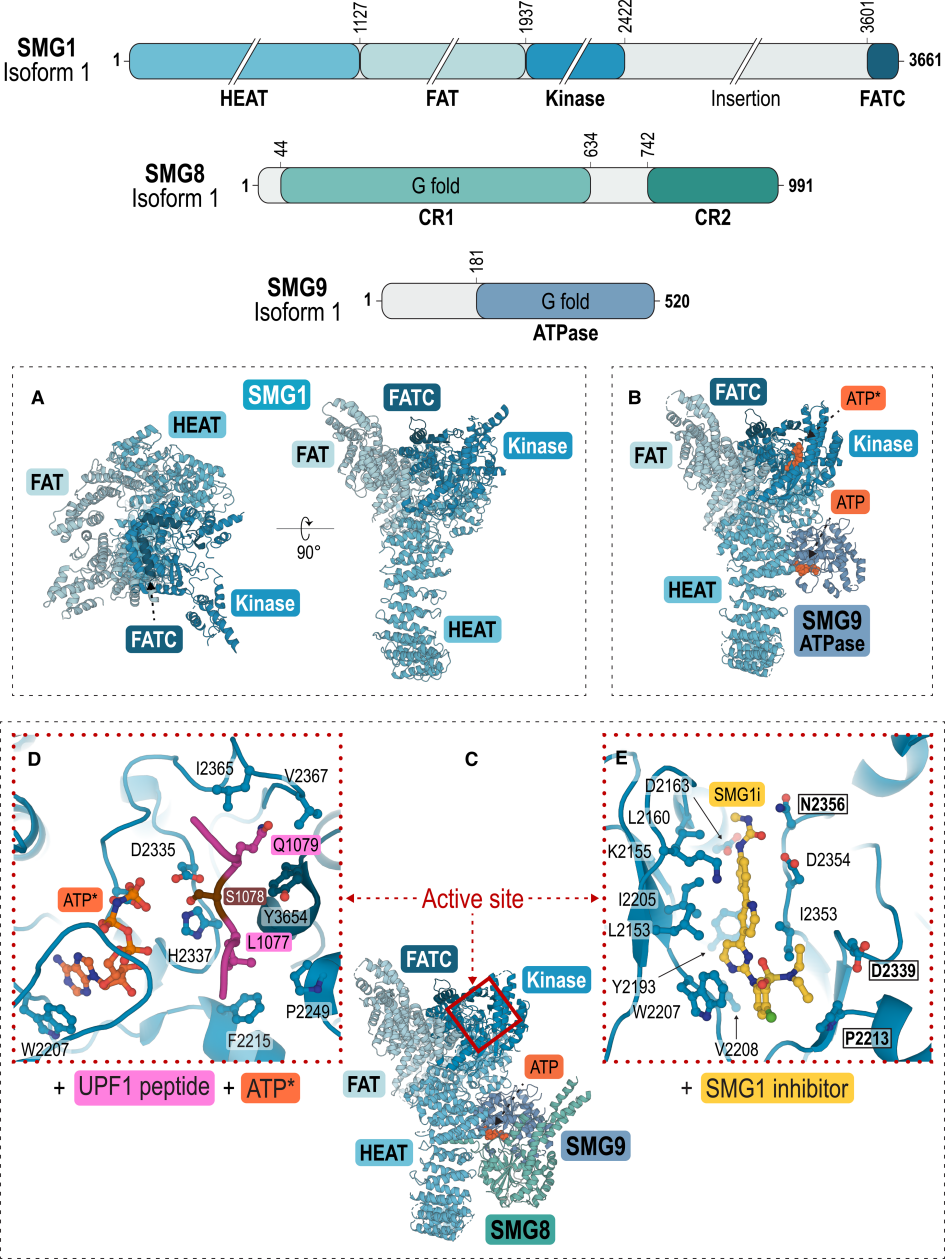
Cryo-EM structures of SMG1–8–9 kinase complexes. Schemes showing the domain architecture of SMG1, SMG8 and SMG9. In the structures, the protein domains are coloured according to their primary structure scheme. (**A**) Top and side view of the structural model of SMG1 kinase with the N-terminal α-helical solenoid arch-containing HEAT repeats (tv-blue) and the globular C-terminus comprised of the FAT domain (light blue), the kinase domain (blue) and the FAT C-terminal (FATC) domain (dark blue) (PDB ID: 6L53) [106]. (**B**) Structural model of SMG1-9 (blue grey) bound with ATP and an ATP analogue (ATP*) (both in orange) (PDB ID: 7PW9) [110]. (**C**) Structural model of SMG1–8–9 bound with ATP (PDB ID: 6SYT) [107]. SMG8 (emerald green) interacts with SMG9. The kinase active site is highlighted with a red box. (**D**) Close-up view of SMG1's active site with a UPF1 leucine-serine-glutamine (LSQ) peptide (in pink) (PDB ID: 6Z3R) [108]. The serine targeted for phosphorylation (S1078) is highlighted in brown. (**E**) Close-up view of SMG1's active site with a pyrimidine derivative inhibitor (SMG1i, yellow) (PDB ID: 7PW4) [110]. Boxes highlight binding pocket residues P2213, D2339 and N2356 which are specific for the SMG1 kinase active site [110].

In metazoans, SMG1 kinase is intimately associated with SMG8 and SMG9, forming the SMG1–8–9 complex (SMG1C) [29]. In SMG1, like in all PIKKs, the kinase domain consists of an N-terminal α-helical solenoid ‘arch’ and a compact C-terminal ‘head’ region (Figure 3A). The N-terminus is formed of (Huntington, Elongation factor 3, A subunit of PP2A, and TOR1) HEAT repeats which interact with SMG8 and SMG9 (Figure 3) [101]. The globular C-terminal ‘head’ region contains a (FRAP, ATM, and TRRAP) FAT domain, the kinase domain, an insertion domain of 1200 residues, and a short FAT C-terminal (FATC) domain (Figure 3) [101,104]. The insertion domain is unique to SMG1 and has been shown to negatively regulate kinase activity in combination with SMG8 and SMG9 (see below) [105].

SMG8 has an N-terminal G-like domain similar to that found in dynamin-like GTPases (Figure 3). It consists of a central eight-stranded β-sheet surrounded by seven α-helices, followed by a bundle of three α-helices to form a stem-like domain [106]. SMG9 contains a similar G-fold domain (Figure 3) which forms a stable pseudo-symmetric heterodimer with SMG8 [106]. SMG9 interacts with SMG1 through an extended coiled α-helical region and a peripheral β-sheet that forms hydrophobic contacts with the HEAT repeats of SMG1 (Figure 3B). Thus, SMG9 bridges SMG8 and SMG1 to form the SMG1C complex (Figure 3C) [29,106–108].

While SMG8 and SMG9 both contain G-like domains, only the SMG9 G-like domain contains a nucleotide-binding site required for NTP hydrolysis [106]. Consistently, a nucleotide bound to SMG9 was observed in crystal and cryo-EM structures [106,107,109]. Ion-pair high-performance liquid chromatography mass spectrometry (HPLC–MS) identified the co-purified nucleotide in SMG9 as ATP in SMG1C [107]. Comparison of the NTP-bound and NDP-bound structures shows a significant difference in the positions of SMG8 and SMG9 in the available structures, with a region of SMG8, most likely derived from the C-terminus, shifting by ∼10 Å and covering the substrate entry channel of SMG1's kinase domain when SMG9 is NTP-bound [106,109]. In agreement with a kinase-inhibitory role for this region, deletion of SMG8's C-terminus resulted in the hyperactivation of SMG1 kinase when compared with the wild-type complex [106]. Notably, the inhibition of SMG1 kinase activity by SMG8's C-terminus seems to be synergistic with the negative regulation by the C-terminal insertion domain of SMG1 [105,106]. Taken together, these findings suggest that UPF1's access to the catalytic site in SMG1C is tightly regulated, and UPF1 phosphorylation occurs after a reorganisation of both the SMG1 C-terminal insertion domain and the SMG8-9 heterodimer [105,106]. This structural rearrangement of SMG1C is suggested to be driven by ATP hydrolysis of SMG9.

UPF1 phosphorylation by SMG1C is the key step in NMD. Recently, Langer and colleagues revealed the molecular basis for phosphorylation site selection by SMG1 kinase by solving the cryo-EM structure of SMG1C with a peptide mimicking a part of UPF1's SQ domain (residues 1074–1084) (Figure 3D) [108]. In the kinase active site of SMG1, Q1079 at position +1 in the UPF1 SQ-domain peptide is positioned in a hydrophobic cage composed of the SMG1 activation segment and the FATC domain (Figure 3D) [108]. In agreement with the importance of Q1079, mutation of this glutamine inhibits phosphorylation. SMG1C's phosphorylation efficiency and specificity are further improved by a hydrophobic residue at position −1 (predominantly leucine) in the UPF1 SQ-domain peptide (Figure 3D) [108]. Taken together, these observations highlight why the LSQ motif found in UPF1 SQ-domain is optimal for binding the SMG1 kinase active site.

A cryo-EM structure of SMG1C in complex with a pyrimidine derivative inhibitor shows this compound bound between the N- and C-terminal lobes of the kinase domain where it interacts with residues P2213 and D2339 in the active site (Figure 3E) [110]. These proline and aspartate residues are unique to SMG1, which may explain the selectivity of the inhibitor for SMG1 compared with other PIKK family members [110]. The SMG1 inhibitor stabilises an autoinhibitory conformation of the SMG1 insertion domain (the 1200-residue region connecting the kinase and FATC domains of SMG1). Based on this finding, the authors suggest that the SMG1 insertion domain contains a PIKK-regulatory domain (PDR) that engages with and occludes access to the kinase active site, in analogy to PDRs identified in other PIKKs. Furthermore, the inhibitory conformation of SMG1's insertion domain is stabilised by interaction with SMG8's C-terminal domain, confirming that these domains work together to down-regulate SMG1 kinase activity [110].

### SMG5, SMG6, SMG7

**Figure 4.**
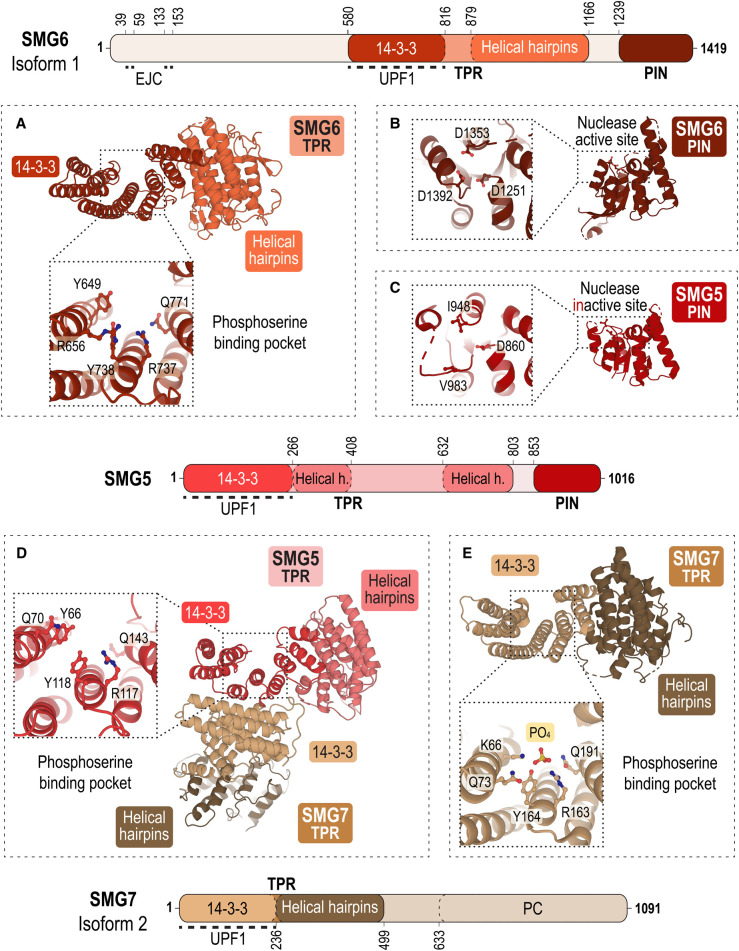
The architecture of mRNA decay factors SMG6, SMG5 and SMG7. Schemes showing the domain architecture of SMG6, SMG5 and SMG7. In the structures, the protein domains are coloured according to their primary structure scheme. (**A**) Structure of the tetratricopeptide (TPR) domain of SMG6 with a 14-3-3-like module (dark red) involved in binding phosphorylated serine/threonine residues of UPF1 and α-helical hairpins (orange) (PDB ID: 4UM2) [71]. The close-up view shows the phosphoserine-binding pocket of SMG6 with key residues highlighted as sticks. (**B**) Structure of SMG6's PIN domain (chocolate) (PDB ID: 2HWW) [115]. The close-up view of the nuclease active site highlights the canonical catalytic triad of acidic residues (D1251, D1353, D1392). (**C**) Structure of SMG5's PIN domain (red) (PDB ID: 2HWY) [115]. The close-up view shows the inactive site in SMG5 comprising only one aspartate (D860, I948, V983). (**D**) Structure of the heterodimer of the TPR domain of SMG5 with a 14-3-3-like module (red) and α-helical hairpins (light red) and the TPR domain of SMG7 (14-3-3-like module, beige; α-helical hairpins, brown) (PDB ID: 3ZHE) [113]. SMG5 and SMG7 interact via their 14-3-3-like modules. The close-up view shows the phosphoserine-binding pocket of SMG5 with key residues highlighted as sticks. (**E**) Structure of the TPR domain of SMG7 with its 14-3-3-like module (beige) and α-helical hairpins (brown) (PDB ID: 1YA0) [116]. The close-up view shows the phosphoserine-binding pocket of SMG7 with key residues and a phosphate ion highlighted as sticks.

Hyperphosphorylated UPF1 recruits decay-inducing factors including SMG5, SMG6 and SMG7 [69,71,72,111,112]. These three proteins are structurally related and have similar tetratricopeptide repeat (TPR) domains, consisting of a 14-3-3-like fold formed by nine antiparallel α-helices, followed by an α-helical domain (Figure 4). [113]. The 14-3-3 fold binds phosphorylated serine/threonine residues (Figure 4A,D,E). However, the three proteins have different C-termini (Figure 4): SMG5 and SMG6 harbour PilT N-terminus (PIN) domains of which only the SMG6 domain has endonuclease activity (Figure 4B,C) [34,35,114,115] whereas SMG7 holds a proline-rich C-terminal (PC) domain [114].

**Figure 5.**
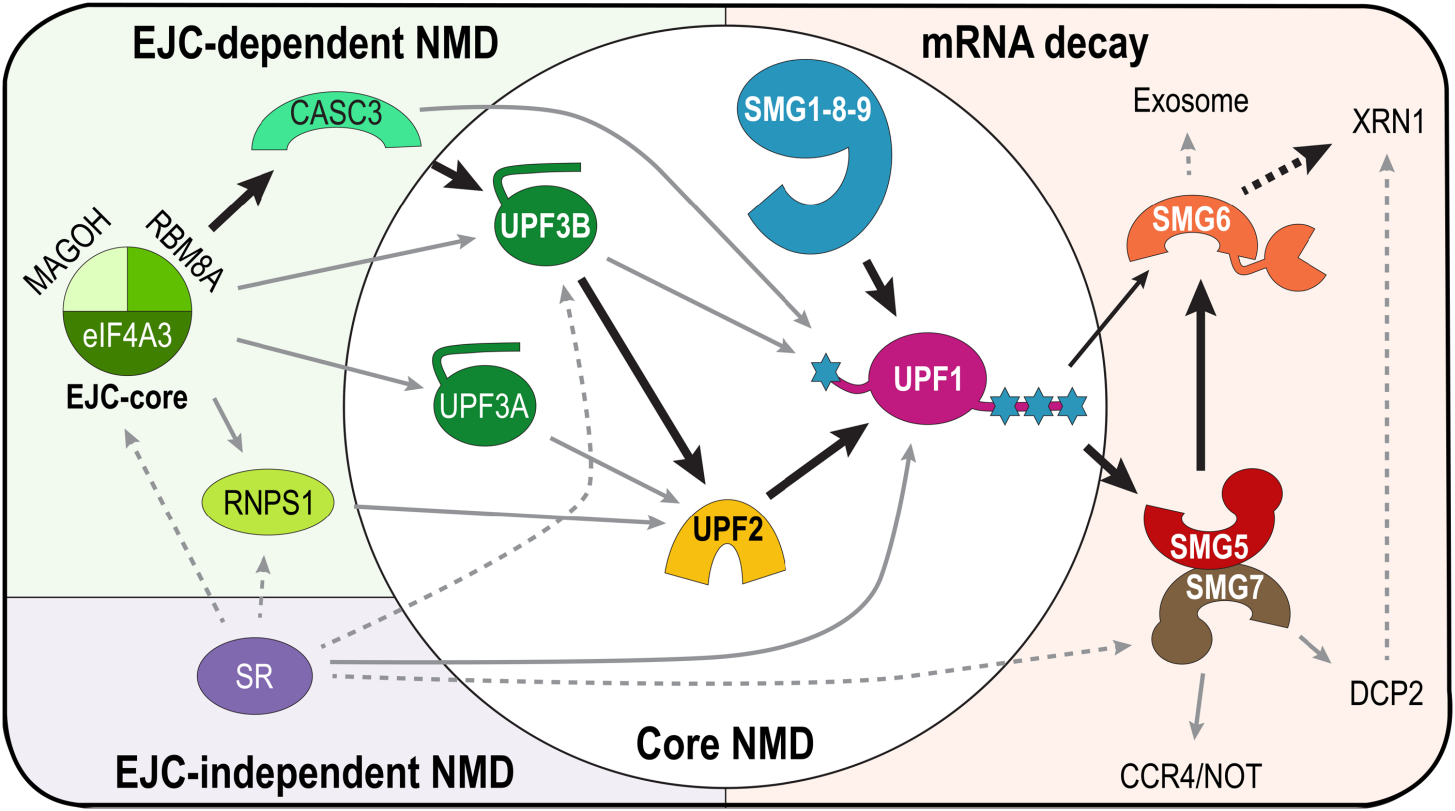
The branched NMD pathways. Scheme summarising the NMD pathway and its branches converging at UPF1 as the key factor. Black thick arrows indicate the canonical NMD pathway in mammalian cells. The EJC-dependent part of the NMD pathway is shown in a green box, the EJC-independent part in a purple box, the core NMD pathway in a white circle and the different mRNA decay routes in an orange box. The branch requiring UPF1, UPF2 and UPF3B presents the canonical NMD branch conserved from yeast to human. UPF3A is an NMD activator in most UPF3B-deficient contexts. In the UPF3-independent NMD branch, RNPS1 stimulates NMD by recruiting UPF2. UPF2-independent NMD is enhanced by CASC3, which binds UPF3B and UPF1. UPF2 activates the SMG1–8–9 kinase complex to phosphorylate UPF1. mRNA decay relies on the recruitment of SMG6 and SMG5–7 by phospho-UPF1. SMG5–7 interact with factors responsible for mRNA deadenylation (CCR4/NOT complex), mRNA 5′-decapping (DCP2), and activates SMG6 endonuclease. Resulting unprotected mRNA fragments are degraded by the exonuclease XRN1 and the exosome complex. SR proteins (in particular SRSF1) enhance NMD by interacting with the EJC-core and its associated proteins as well as NMD factors. SR proteins can also promote mRNA decay in an EJC-independent manner.

The structure of the SMG5–7 TPR domains shows a heterodimer with an evolutionarily conserved interface between the two 14-3-3-like folds (Figure 4D) [113,116]. Complex formation with phospho-UPF1 leads to the recruitment of the CCR4–NOT deadenylation complex [117]. Deadenylation of the target mRNA is followed by mRNA decapping and 5′–3′ and/or 3′–5′ exonucleolytic degradation (Figure 5) [114,117].

In contrast with SMG5–7, SMG6 is a monomer, consisting of a 14-3-3-like fold domain, an EJC-binding domain, and a PIN domain with endonuclease activity (Figure 4A,B) [34,48,71,115]. SMG6 has been shown to cleave mRNA close to the PTC, followed by 5′–3′ degradation via the exonuclease XRN1 and 3′–5′ degradation by the exosome complex [35,37,38,118,119]. Co-IP assays showed that SMG6 associates via its two EBMs with MAGOH–RBM8A, competing with UPF3B for EJC-binding [48], and CASC3 was shown to stimulate SMG6 endonuclease activity [47]. In conclusion, the interaction of SMG6 with the EJC is suggested to facilitate its recruitment to nonsense mRNA and enzymatic activation [48].

SMG5 and SMG7 complementation experiments in knockout cells indicate that the SMG5–7 heterodimer is essential for SMG6's endonuclease activity [120]. SMG6 was unable to cleave nonsense mRNAs in the absence of SMG5–7, but a transient interaction between SMG6 and UPF1 could be detected in TurboID experiments nevertheless [120]. This finding agrees with pulldown assays showing that SMG6 can interact also with unphosphorylated UPF1 [71]. While previous work suggested that SMG6 and SMG5–7 promote the degradation of NMD targets in a redundant manner — either by endonucleolytic cleavage or by deadenylation and decapping (Figure 5) [113,121,122] — the work from Boehm and colleagues emphasises the interdependence of SMG6- and SMG5–7-mediated degradation paths [120]. The authors suggest a model, where SMG5–7 recruitment to phospho-UPF1 represents an authorising step required for SMG6-mediated degradation of the target transcript.

## Mammalian NMD is branched and highly specialised

In mammalian cells, NMD functions extend beyond quality control of protein synthesis. Illustrative of this, NMD is a major contributor to mammalian gene regulation by targeting ∼10% of the transcripts [3,5,8,123]. Endogenous transcripts recognised by the NMD machinery are typically characterised by a long 3′-UTR, a 3′-UTR intron, or a short upstream open reading frame (ORF) in the 5′-UTR [3,5,124–126]. Moreover, NMD targets mRNAs encoding selenoproteins, small nucleolar RNAs and long non-coding RNAs [3,5,38,127]. This wide range of NMD substrates implies that more specific mechanisms have evolved to allow the NMD machinery to accurately select target RNAs, while having a highly regulated and fine-tuned activity. Consequently, mammalian NMD is not one pathway but specialised and subdivided into several branches. All NMD pathways share a common feature, namely the central role of UPF1 (reviewed in *e.g.* [5,128]) (Figure 5). The canonical NMD mechanism is EJC-dependent, involving the formation of the SURF and DECID complexes (see above). Furthermore, EJC-dependent NMD can branch off into UPF2-independent or UPF3B-independent pathways [58,121,129], and transcripts with long 3′-UTRs are degraded in an EJC-independent manner, involving UPF1, SMG1 as well as UPF2 and/or UPF3B [13,121].

In EJC-dependent NMD, further sub-branches exist due to varying EJC composition resulting from the presence of mutually exclusive factors (Figure 5) [39]. (i) In the presence of EJCs containing RNPS1, NMD has been shown to occur independently of UPF3B [58]. In this branch of NMD, the RNSP1-containing EJC can directly recruit UPF2 and elicit NMD (Figure 5). (ii) Inversely, UPF2-independent NMD can occur when CASC3 is bound in the EJC. CASC3 binds eIF4A3, UPF3B and UPF1, and UPF3B was shown to interact directly with UPF1 and eRF3a [39,59,91]. Therefore, CASC3 could enhance the interaction of UPF3B-bound EJC with UPF1 and thus promote NMD in the absence of UPF2 (Figure 5) [58]. Even though several studies support this CASC3-enhanced model [39,47,59], the mechanisms underlying the stimulation of UPF1's ATPase and helicase functions and downstream NMD activation in the absence of UPF2 remain unclear to date.

RNPS1- and CASC3-enhanced NMD pathways differ in the subcellular localisation and lifetime of the EJCs associated with these factors [39]. RNPS1 binds to mRNAs during co-transcriptional splicing, therefore RNSP1-containing EJCs are enriched in the nucleus and at early cytoplasmic EJC stages. In contrast, CASC3 engages with the EJC post-splicing following an EJC-compositional switch which occurs either before or during translation in the cytosol at later EJC stages [39,59]. In agreement, RNPS1-enhanced UPF3B-independent NMD is suggested to target mRNAs translated shortly after their export from the nucleus, directly in the nuclear periphery, while CASC3-enhanced UPF2-independent NMD could target mRNAs anytime during translation [39,58].

Splicing and NMD are linked in EJC-dependent NMD, and members of the serine- and arginine-rich (SR) protein family of splicing factors, which bind nascent mRNAs during co-transcriptional splicing, are suggested to enhance NMD [130–132]. Consistently, SR Splicing Factors 1 and 3 (SRSF1, SRSF3) strongly promote NMD when they are bound or tethered to a mRNA downstream of a PTC [131]. SRSF1 interacts in an RNA-independent manner with EJC-core components (eIF4A3, MAGOH and RBM8A), as well as other EJC-associated proteins including RNPS1 and UPF3B, but not CASC3 or UPF2 (Figure 5) [131]. However, SRSF1 interaction with the EJC is not required for its NMD-enhancing function [131]. Rather, SRSF1 interacts directly with UPF1 and appears to recruit UPF1 to the mRNA in the nucleus, and this process has been suggested to be responsible for SRSF1-enhanced NMD (Figure 5). Furthermore, recent reports suggest that in the cytoplasm, SRSF1 has an additional NMD-enhancing activity because co-IP assays show an interaction between SRSF1 and serine/threonine-protein phosphatase 2A (PP2A) and SMG7 [131,133]. Via these interactions, SRSF1 may promote the dephosphorylation of UPF1, which would result in the release of UPF1 from SMG6 and SMG5–7. Following on from this, the NMD factors are then recycled and ready for a new round of NMD [131,134,135]. The ability of SRSF1 to enhance NMD of intron-less transcripts when tethered downstream of a PTC supports the existence of an EJC-independent pathway in mammals, as it was suggested for long 3′-UTR transcripts [13,131].

Knock-down of several NMD factors showed that UPF1 and SMG1 are strictly required for EJC-independent NMD, while UPF3B or UPF2 can only be depleted separately [121], confirming their role of core NMD factors (Figure 5). The existence of different branches of NMD underscores a sophisticated fine-tuning of NMD for the regulation of gene expression in mammalian cells in a context-dependent manner. Examples for localised NMD responses have been reported for dendritic and axonal mRNAs in neurons and for mRNAs localised at the endoplasmic reticulum (ER) [136]. The ER is specialised for the translation and translocation of secreted and integral membrane proteins while also being the site of the unfolded protein response (UPR) [137,138]. Immunofluorescence microscopy indicates that UPF3B and SMG6 co-localise with the ER chaperone BiP and thus reside at the ER, and SMG6 is up-regulated upon induction of ER-stress [139]. *Vice versa*, NMD is involved in regulating the expression of UPR factors, including ATF-3, ATF-4, PERK and IRE1α [140,141]. Genome-wide RNA interference screens identified the neuroblastoma-amplified sequence protein (NBAS) and the helicase DHX34 as additional NMD factors. These proteins were found to regulate the stability of a subset of mRNAs encoding proteins predominantly involved in the response to cellular and environmental stress as well as NMD factors including UPF1 [136,141,142]. Previous reports show that NMD is inhibited by cellular stress, including amino acid starvation, up-regulation of the UPR and hypoxia [143]. More recently, NBAS was shown to bind mRNA and to interact with UPF1, SMG6 and SMG5–7 [136]. In addition, NBAS knockdown leads to a five-fold increase in membrane-associated mRNAs, in agreement with a role of NBAS at the ER [136]. Based on these findings, NBAS is suggested to recruit UPF1 to the ER membrane to facilitate an ER-associated NMD pathway. Taken together, NMD appears to respond to cellular stress and to be part of a feedback loop that activates the cellular stress response.

Different versions of the NMD core factors are used in context- and cell type-specific manners. The paralogs UPF3A and UPF3B are one example. Despite their homology, UPF3A is rapidly degraded in the cell and requires UPF2-binding for its stabilisation [95]. In most tissues, UPF3B is significantly more abundant than UPF3A, with one exception being the adult testis [98]. In an UPF3B-deficient context (see above), UPF3A is stabilised and activates NMD [50,51,97]. However, during male germ cell development, the X-linked *Upf3B* gene is transcriptionally silenced, due to meiotic sex cell chromosome inactivation, such that UPF3A is highly expressed [98]. Surprisingly, NMD was enhanced when *Upf3A* was removed from developing male germ cells, suggesting that UPF3A is a NMD repressor in these cells [98]. The presence of UPF3A and the absence of UPF3B was shown to stabilise mRNAs encoding proteins which are essential for spermatogenesis and male fertility [98]. At the same time, normal levels of NMD targeting other mRNAs with long 3′-UTRs were detected in UPF3A/3B-deficient cells, indicating down-regulation of one NMD branch in male germ cells [98,144].

A further example of branched NMD is UPF1 which exists in two splice variants in mammalian cells, UPF1_LL_ and UPF1_SL_, with different functional roles and outcomes for NMD (see above). These variants are differentially expressed in human tissues: while UPF1_LL_ represents ∼20% of expressed UPF1 in most tissues, it is up-regulated to ∼35% in muscles and down-regulated to ∼15% in the liver [81]. The specific knockdown of UPF1_LL_ indicates that it participates in NMD under normal cellular conditions. Transcriptome analyses revealed that UPF1_LL_ NMD targets were enriched in mRNAs encoding proteins preferentially translated at the ER, such as integral membrane proteins [81]. Interestingly, during ER stress, these UPF1_LL_ NMD targets remained down-regulated. In contrast, mRNAs preferentially targeted by UPF1_SL_ were up-regulated in agreement with stress-induced NMD inhibition [81]. This differential regulation of NMD is suggested to result from the down-regulation of translation which is induced by the integrated stress response and mediated by the phosphorylation of eIF2α. Similarly, a moderate slowdown of translation by treating cells with sub-inhibitory concentrations of the antibiotics Blasticidin S or cycloheximide was reported to repress NMD and resulted in higher expression levels of the protein encoded by the nonsense mRNA [145]. In this context, UPF1_LL_ could detect rare aberrant translation termination events and induce NMD, possibly due to its increased residence time on mRNAs compared with UPF1_SL_ [81].

## Viral evasion of NMD

Beyond translational control, NMD targets viral RNA transcripts and genomes, which often contain unique features that could elicit an NMD response. Accordingly, NMD plays a crucial role in controlling viral pathogen replication in both plants and animals [146–149]. Because of this, viruses have expanded their repertoire for infecting hosts by evolving new mechanisms for evading and inhibiting NMD. Research into these viral processes has provided vital new understandings into the complex protein–protein and RNA–protein interactions during NMD and highlighted two strategies employed by viruses to avoid NMD, known as *cis* and *trans* methods (reviewed *e.g.* in [150]). *Cis* methods are characterised by features of the viral RNA that either directly prevent NMD or recruit host factors that antagonise NMD. *Trans* methods are based on proteins encoded by the virus that are synthesised to directly interfere with NMD leading to a simultaneous up-regulation of host cell nonsense mRNAs [150].

An example for a *cis*-based mechanism is found in the Rous sarcoma virus (RSV). The retroviral mRNA of RSV contains an RNA stability element (RSE) which prevents UPF1 from initiating NMD. The RSE contains multiple polypyrimidine tracts which recruit PTBP1 (see above) [151,152]. PTBP1 prevents the translocation of UPF1 by acting as a physical barrier on the RNA promoting the dissociation of UPF1 from the RNA [83].

An example for a *trans*-acting mechanism is found in the human T-cell leukaemia virus type I (HTLV-1). HTLV-1 is a positive-sense RNA retrovirus that randomly integrates its genetic material with the host genome, causing aggressive adult T-cell leukaemia. The HTLV-1 genome contains >10 ORFs which are regulated by alternative splicing and programmed ribosomal frameshifting. The mRNAs of HTLV-1 should be targets for NMD due to various features including long 3′-UTRs of ∼4000 bases. However, HTLV-1 expresses two positive *trans*-regulatory proteins, Rex and Tax, which aid in NMD evasion [153–156]. Co-IP experiments against Tax from HTLV-1-infected cells, identified INT6 (one of the thirteen eIF3 subunits) and UPF1 as interaction partners [156]. NMD inhibition by Tax depends on its interaction with INT6, as Tax mutants that are unable to bind to INT6 were found to have no effect on NMD [157,158], suggesting an involvement of eIF3 in NMD of mRNAs with long 3′-UTRs. In addition, Tax interacts with the helicase domain of UPF1 and reduces its RNA-binding affinity by blocking the RNA entry site. Using single-molecule techniques, Tax was also shown to interact with RNA-bound UPF1, blocking its ATPase and helicase activities leading to the dissociation of UPF1 from the mRNA [156,158]. Taken together, Tax appears to inhibit NMD by binding UPF1 and INT6 and interfering with their interaction [156]. Co-IPs additionally indicate that HTLV-1 RNA is still able to interact with phosphorylated UPF1 which accumulates in P-bodies for degradation. P-bodies are cytoplasmic ribonucleoprotein granules that consist mostly of proteins associated with mRNA decay and translationally repressed proteins. When NMD was inhibited, an increased accumulation of UPF1 in P-bodies was observed [156,157]. In this inhibited NMD context, Tax was found to co-localise with UPF1 in P-bodies causing its hyperphosphorylation and leading to a decrease in free unphosphorylated UPF1 and further inhibition of NMD [157,159]. Thus, while Tax protects its encoding RNA from NMD, it also subjects the host's gene expression to dysregulation by stabilising endogenous NMD targets through global NMD inhibition [156,157].

The strategies deployed by viruses to evade NMD continue to be a rich field of discovery providing important new insights into basic NMD mechanisms as well as potential antiviral treatment strategies.

## Concluding remarks

Research into NMD using a diverse set of model systems suggests that the NMD pathway has acquired an increasing importance over the course of evolution. Although *Saccharomyces cerevisiae* and *Caenorhabditis elegans* are viable without a functional NMD pathway [160,161], NMD is essential for viability in vertebrates [162–164]. This gain of importance with increasing organism complexity corroborates the expansion of NMD functions beyond translational control spanning into antiviral defence and, importantly, the global regulation of gene expression. In mammalian cells, the existence of multiple NMD branches expanded by factor variants and localised functions emerge, each acting on specific subsets of mRNA targets with fine-tuned efficiency, depending on the subcellar localisation, the tissue and cell types, as well as the developmental stage.

These diverse and vital roles played by NMD are highlighted by the medical importance of NMD, as ∼20% of human genetic diseases caused by single base pair mutations are related to NMD [165]. To develop new therapeutic strategies, an advanced understanding of the molecular mechanisms of NMD is needed, including high-resolution structures of complexes to provide insights into the interplay of NMD factors, and their interactions with the translation machinery and the EJC. In fact, crucial basic questions remain unanswered such as how a translation termination event is recognised as aberrant, how recruitment of NMD factors to target mRNAs is achieved, and what is their function in the different NMD branches. Future research in the NMD field will help in elucidating the molecular basis of the cross-talk between the NMD machinery and quality control mechanisms regulating cellular homeostasis, including ribosome quality control and the cellular stress response.

## Abbreviations

ADP: adenosine diphosphate
ATM: ataxia telangiectasia mutated
EBM: EJC-binding motif
EJC: exon-junction complex
ER: endoplasmic reticulum
HTLV-1: human T-cell leukaemia virus type I
LSQ: leucine-serine-glutamine
NBAS: neuroblastoma-amplified sequence protein
NMD: nonsense-mediated messenger RNA decay
PIKKs: phosphatidylinositol-3-kinase-related kinases
PTC: premature termination codon
RBD: RNA-binding-like domain
RRM: RNA Recognition Motif
RSE: RNA stability element
RSV: Rous sarcoma virus
SMG1C: SMG1–8–9 complex
TPR: tetratricopeptide repeat
UPF: up-frameshift protein
UPR: unfolded protein response
